# Cytoplasmic Polyadenylation Element Binding Protein Deficiency Stimulates PTEN and Stat3 mRNA Translation and Induces Hepatic Insulin Resistance

**DOI:** 10.1371/journal.pgen.1002457

**Published:** 2012-01-12

**Authors:** Ilya M. Alexandrov, Maria Ivshina, Dae Young Jung, Randall Friedline, Hwi Jin Ko, Mei Xu, Bryan O'Sullivan-Murphy, Rita Bortell, Yen-Tsung Huang, Fumihiko Urano, Jason K. Kim, Joel D. Richter

**Affiliations:** 1 Program in Molecular Medicine, University of Massachusetts Medical School, Worcester, Massachusetts, United States of America; 2 Research Computing, Information Service Department, University of Massachusetts Medical School, Worcester, Massachusetts, United States of America; 3 Program in Gene Function and Expression, University of Massachusetts Medical School, Worcester, Massachusetts, United States of America; 4 Departments of Epidemiology and Biostatistics, Harvard University, Boston, Massachusetts, United States of America; 5 Department of Medicine, Division of Endocrinology, Metabolism, and Diabetes, University of Massachusetts Medical School, Worcester, Massachusetts, United States of America; Kobe University Graduate School of Medicine, Japan

## Abstract

The cytoplasmic polyadenylation element binding protein CPEB1 (CPEB) regulates germ cell development, synaptic plasticity, and cellular senescence. A microarray analysis of mRNAs regulated by CPEB unexpectedly showed that several encoded proteins are involved in insulin signaling. An investigation of *Cpeb1* knockout mice revealed that the expression of two particular negative regulators of insulin action, PTEN and Stat3, were aberrantly increased. Insulin signaling to Akt was attenuated in livers of CPEB–deficient mice, suggesting that they might be defective in regulating glucose homeostasis. Indeed, when the *Cpeb1* knockout mice were fed a high-fat diet, their livers became insulin-resistant. Analysis of HepG2 cells, a human liver cell line, depleted of CPEB demonstrated that this protein directly regulates the translation of PTEN and Stat3 mRNAs. Our results show that CPEB regulated translation is a key process involved in insulin signaling.

## Author Summary

One major hallmark of diabetes is insulin resistance in peripheral tissues that is controlled at the posttranslational level. For example, insulin activates a kinase cascade that leads to the phosphorylation of Akt, a centrally important molecule that regulates glucose metabolism. In this study, we define a translational regulatory pathway that mediates insulin action in the liver. The Cytoplasmic Polyadenylation Binding Protein (CPEB) interacts with mRNA to control translation; knockout mice that lack CPEB exhibit high-fat-diet-induced liver insulin resistance and do so by having aberrant expression of major insulin signaling molecules, in particular PTEN 3 and Stat3. Our data further suggest that CPEB modulates, in a manner similar to a rheostat, functionally related mRNAs that encode proteins involved in insulin signaling.

## Introduction

The maintenance of glucose homeostasis requires that organisms respond to changing environmental conditions by balancing pancreatic insulin secretion with the ability of tissues, particularly liver, muscle, and fat, to respond to hormone-induced signaling by importing or secreting glucose [1], [2]. Obesity and other insults that promote diabetes do so by causing inflammation and insulin resistance, which is characterized by the inability of tissues to transduce insulin/insulin receptor interactions into elevated glucose uptake in muscle and adipose tissue and/or efficient insulin-mediated suppression of gluconeogenesis in liver. In peripheral tissues such as those noted above, insulin association with the insulin receptor (InsR; NM_010568.2) induces insulin receptor substrates (IRS1/2; NM_010570/NM_001081212.1) tyrosine phosphorylation, which through activated PI3-kinase, promotes the phosphorylation of phosphatidylinositol 4,5-bisphosphate (PIP2) to yield phosphatidylinositol (3,4,5)-trisphosphate (PIP3). This process is inhibited by the phosphatase PTEN (NM_008960.2). PIP3 promotes the membrane localization of PDK1 (NM_008960.2) and Akt1 (NM_011062.4), where PDK1 and PDK2 phosphorylate Akt1, resulting in glucose uptake and metabolism. In addition to PTEN, a number of factors can regulate these events. For example, inflammatory pathways have a profound negative impact on insulin signaling; in one instance, Stat3 activation induces Socs3 transcription, which in turn indirectly represses InsR and IRS1/2 activities [3], [4].

CPEB (NM_007755.4) is an mRNA binding protein that controls cytoplasmic polyadenylation-induced translation by interacting with the 3′ UTR cis-acting cytoplasmic polyadenylation element (CPE) and three regulatory proteins including Gld2 (NM_001094423.1), a poly(A) polymerase; PARN (NM_001087674.1), a deadenylating enzyme, and Maskin (NM_001088495.1), which also associates with eIF4E (NM_001090548.1), the cap-binding factor [5]. Initial CPEB-repression occurs because Maskin binding to eIF4E precludes the eIF4E-eIF4G interaction that is necessary to recruit the 40S ribosomal unit to the 5′ end of the mRNA. Because Maskin is tethered to CPE, only CPE-containing RNAs are repressed. CPEB-induced stimulated (i.e., deprepressed) translation is initiated when CPEB is phosphorylated on S174 or T171 (species-dependent), which expels PARN from the ribonucleoprotein complex. This event allows Gld2 to polyadenylate the RNA by default. The newly elongated poly(A) tail is then bound by poly(A) binding protein (PABP), which also binds eIF4G; the PABP/eIF4G complex displaces Maskin from eIF4E, allowing for formation of the initiation complex on the 5′ cap structure.

By regulating mRNA-specific translation, CPEB profoundly influences gametogenesis and early development [6], [7], [8], neuronal synaptic plasticity [9], [10], and cell-cycle progression [11]. CPEB also regulates translation during cellular senescence; fibroblasts derived from *Cpeb1* knockout (KO) mice do not senesce as do wild type (WT) cells, but are immortal [12]. Similarly, human cells depleted of CPEB bypass senescence and have a three-fold extended lifespan [13]. In the mouse cells, aberrant over-expression of myc (NM_001177353.1) mRNA is at least one event that mediates the senescence response [12], while in human cells, reduced p53 (NM_000546.4) mRNA translation accounts for the senescence bypass [13].

To identify additional mRNAs that are translationally regulated by CPEB during senescence, we used sucrose density gradient centrifugation to prepare polysomes from WT and *Cpeb1* KO mouse embryo fibroblasts (MEFs) followed by microarray analysis. Unexpectedly, several mRNAs encoding insulin-signaling molecules (see below) were found to be excessively polysomal in the KO versus WT MEFs. Consequently, we investigated the involvement of CPEB in insulin signaling and glucose homeostasis. In *Cpeb1* KO liver, muscle, and fat, there was a dramatic and widespread mis-expression of insulin-signaling proteins. Increased expression of two major negative regulators of insulin signaling, PTEN and Stat3 (NM_213659.2), was associated with reduced Akt phosphorylation in both *Cpeb1* KO liver and in CPEB-depleted HepG2 human liver cells. When fed on a high fat diet, WT and *Cpeb1* KO mice were both obese, but only the KO animals displayed liver insulin resistance. These and other data demonstrate that CPEB control of PTEN and Stat3 mRNA translation is essential for liver insulin signaling and glucose homeostasis.

## Results

### CPEB deficiency results in widespread impairment of insulin signaling

**Figure 1:**
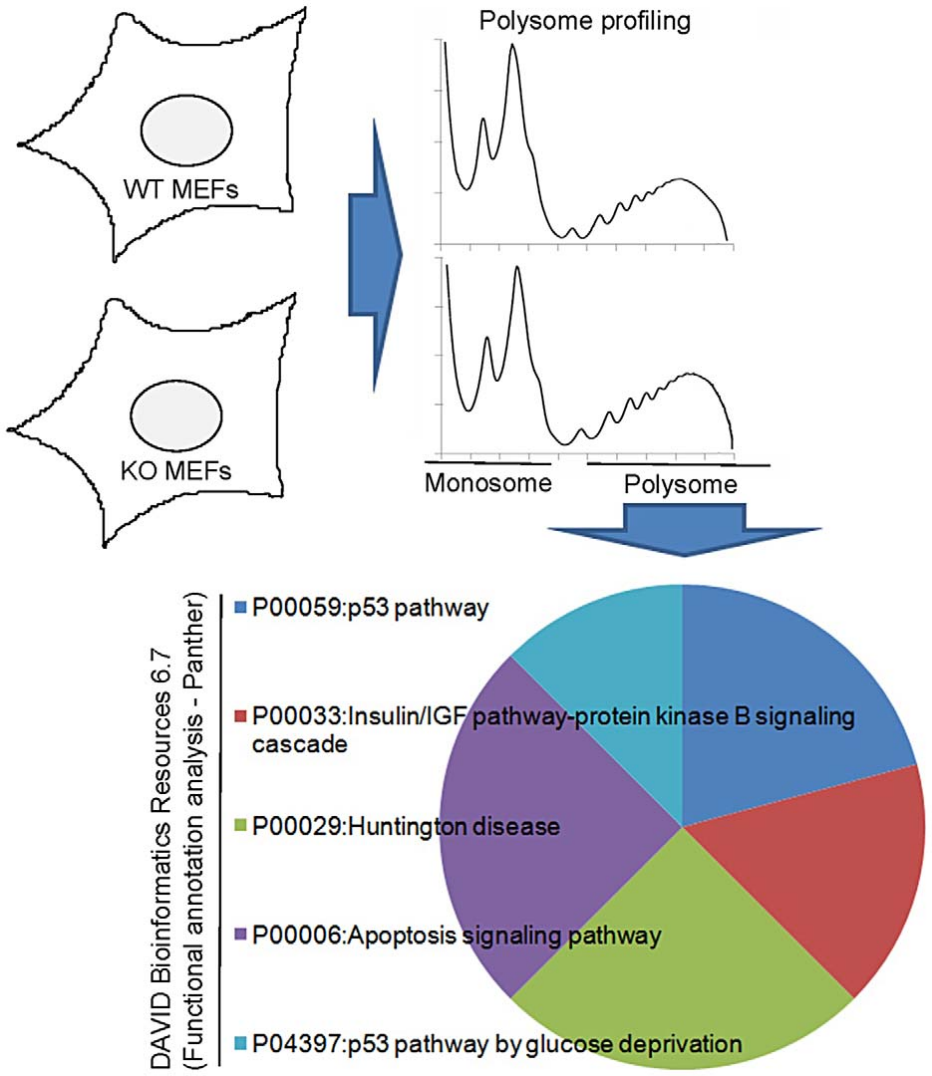
Analysis of polysome sucrose gradients reveals CPEB control of insulin signaling. Extracts prepared from WT and CPEB KO MEFS were centrifuged through sucrose gradients; the fractions containing polysomes were pooled, the RNA extracted, and used to probe microarrays. A functional annotation analysis reveals changes in the translation of a number of mRNAs involved in insulin signaling.

**Figure 2:**
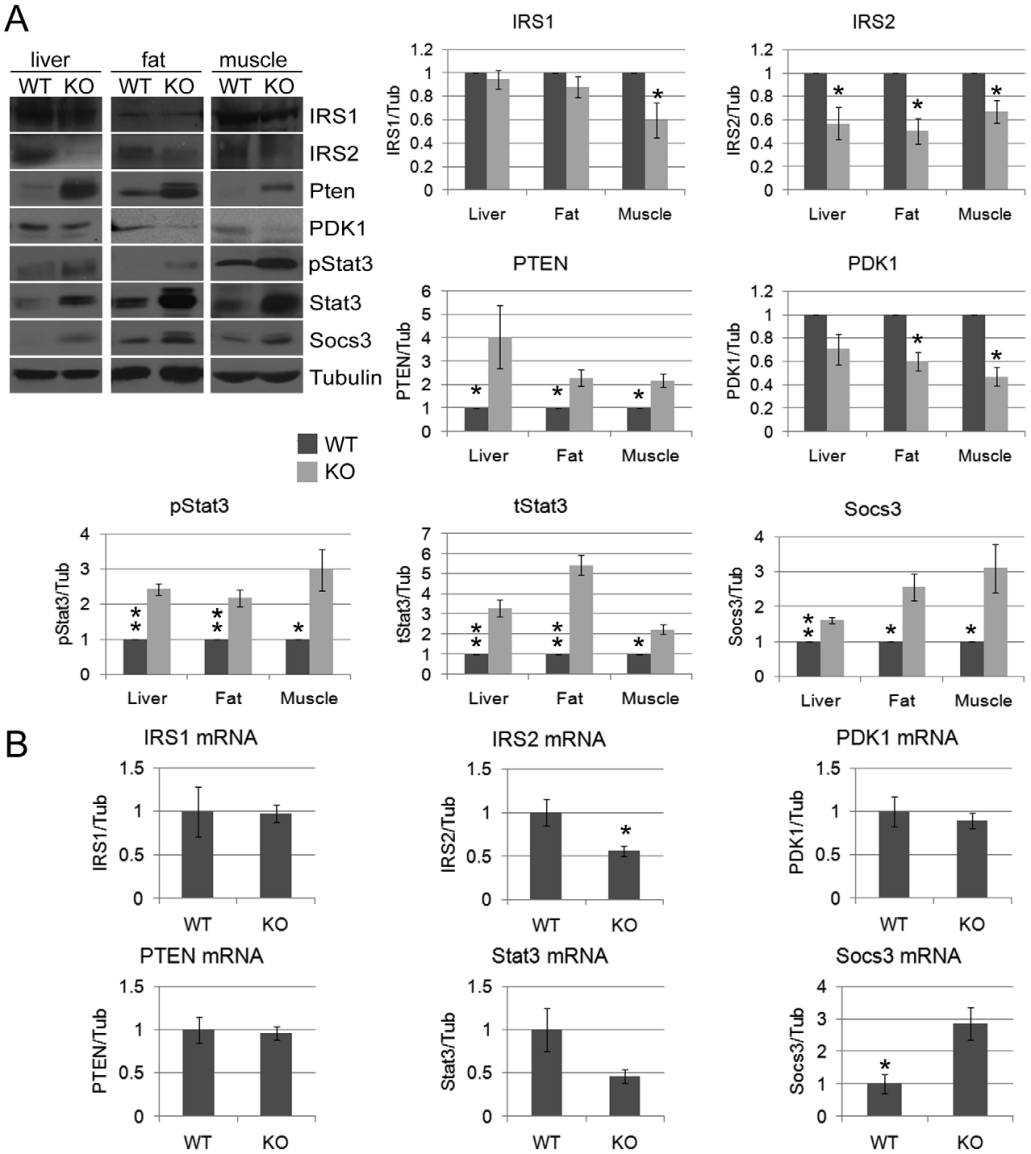
Dramatic and widespread changes in insulin signaling molecules in CPEB knockout mice. (A) Western blot analysis and quantification of IRS1, IRS2, PTEN, PDK1, phospho-Stat3 (S727), total Stat3, Socs3, and tubulin as a loading control from WT and CPEB knockout liver, fat, and muscle. (B) Quantitative RT-PCR analysis of mRNAs encoding IRS1, IRS2, PTEN, PDK1, Stat3, and Socs3 mRNAs from WT and *Cpeb1* KO liver. Data are represented as mean +/− SEM. At least 3 animals per group were used for the experiment. Asterisks refer to statistical significance at the p<0.05 (*) or p<0.01 (**) levels (Student's t test).

**Figure 3:**
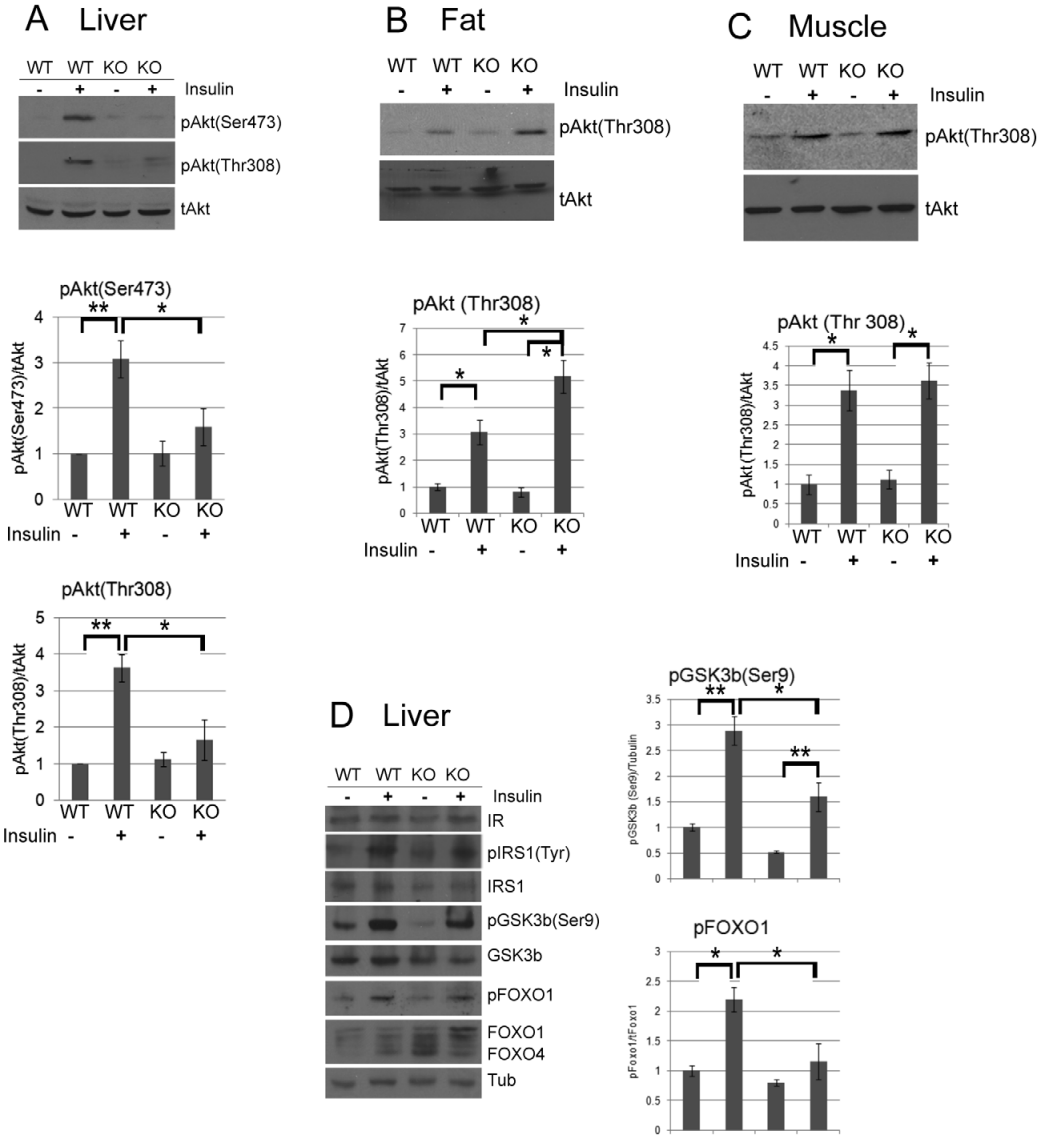
CPEB mediates insulin signaling in the liver. (A) Western blot and quantification of total and phospho-Akt (serine 473 and threonine 308) from liver of WT and *Cpeb1* KO mice, some of which were injected with insulin. The pAkt (Ser473) and pAkt (Thr308) data were analyzed with ANOVA with (p<0.05, *; p<0.01, **). Data are represented as mean +/− SEM. In this and all panels, at least 3 animals per group were used for the experiment. (B,C) Phospho-Akt (threonine 308) in CPEB KO fat and muscle, respectively. Analysis as in panel A. (D) Examination of insulin signaling molecules in WT and CPEB KO liver. Analysis as in panel A.

MEFs derived from CPEB knockout mice do not senesce as do WT MEFs, but instead are immortal [12]. Similarly, primary human skin fibroblasts in which CPEB is depleted also bypass senescence [13], [31]. Senescence bypass is caused by altered translation of myc and p53 mRNAs, which are directly bound by CPEB. We suspected that additional mRNAs whose translation is controlled by CPEB would also be involved in senescence; to identify them, we performed microarray analysis of mRNAs associated with polysomes in wild type (WT) and *Cpeb1* KO MEFs (Figure 1; Table S1). Several of these mistranslated mRNAs control p53-related processes, which given the importance of p53 in senescence, was not unexpected result. However, we also noticed that several aberrantly translated mRNAs encoded proteins involved in insulin signaling, which surprisingly suggested that this signal transduction pathway and perhaps glucose metabolism might be compromised in *Cpeb1* KO mice. Consequently, we probed western blots for insulin signaling proteins derived from WT and *Cpeb1* KO liver, muscle, and fat. Figure 2A shows dramatic and widespread mis-expression of many insulin-signaling molecules (reference 8 shows knockout of CPEB gene; Figure S1A shows a western blot of CPEB in WT and KO liver). Critical proteins such as PTEN, Stat3, and Socs3 (NM_007707.3) were all elevated in KO liver, muscle, and fat, usually by 2–3 fold. With the exception of Socs3 and IRS2 mRNAs, the levels for the mRNAs encoding these proteins were rarely changed in liver. (Figure 2B). One central insulin-signaling molecule is Akt, which is phosphorylated on S473 and T308 following insulin stimulation; the phosphorylation of these residues is necessary to maintain glucose homeostasis. In fasting WT mice injected with insulin, there was a dramatic increase in liver phospho-S473 and phospho-T308 Akt, but not total Akt, as expected. In *Cpeb1* KO mice, however, there was virtually no phosphorylation of Akt irrespective of insulin treatment (Figure 3A). In both fat (Figure 3B) and muscle (Figure 3C), AKT was phosphorylated in the KO in response to insulin, indicating that liver is uniquely affected by the lack of CPEB. In the KO liver, phospho-GSK-3β is elevated upon insulin stimulation, albeit not as much as WT (Figure 3D). Phospho-FOXO-1 was also somewhat elevated in KO liver in response to insulin, but significantly less than in WT. In muscle and fat, these and other signaling molecules were largely unaltered by insulin treatment (Figure S1B). It is curious why these two tissues, but not liver, had normal levels of signaling molecules following application of insulin. Perhaps tissue-specific regulatory molecules such as kinases or miRNAs ameliorate or compensate for the lack of CPEB. Taken together, these data indicate that insulin signaling, particularly in the liver, is compromised by CPEB depletion.

**Figure 4:**
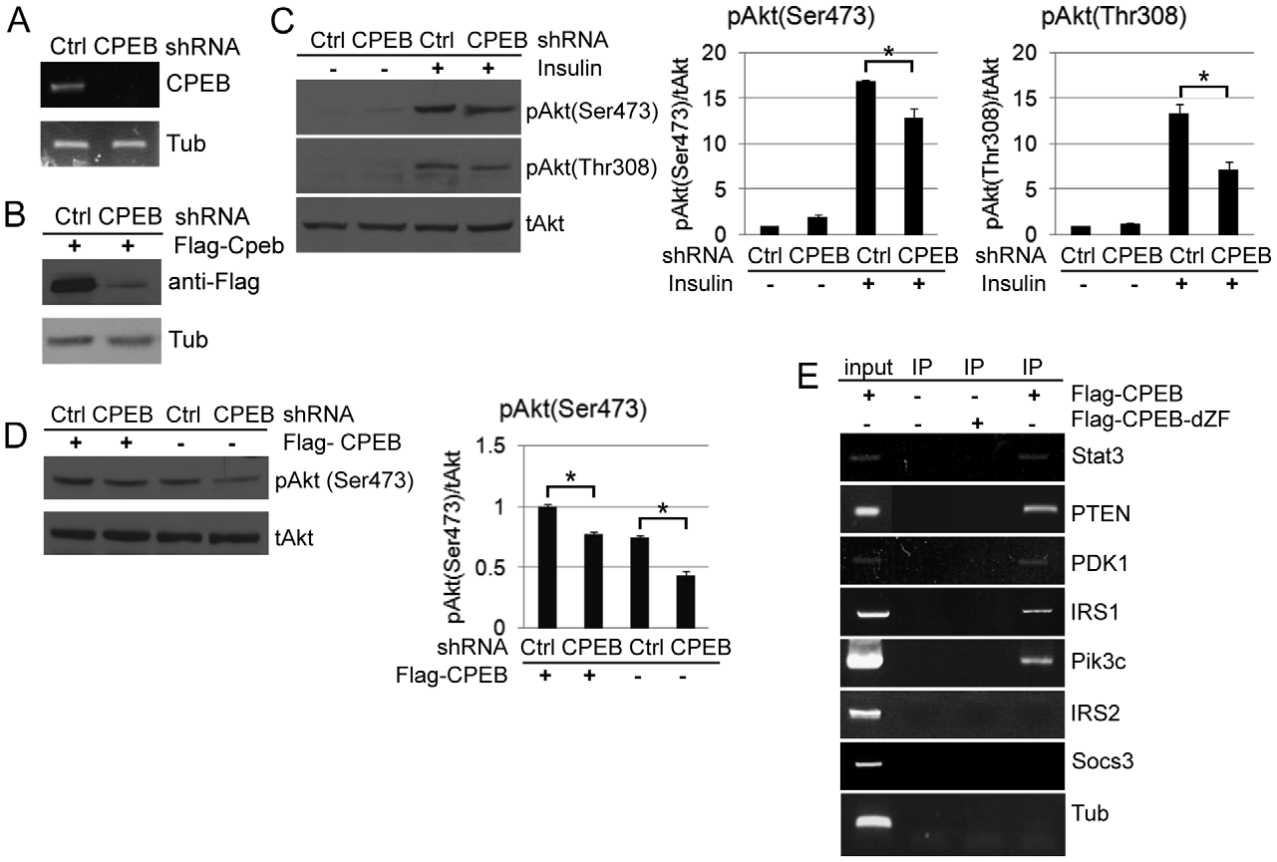
CPEB depletion in HepG2 cells results in aberrant insulin signaling. (A) HepG2 cells were infected with lentiviruses expressing a control shRNA or one directed against CPEB; extracted RNA was then assessed for CPEB RNA, and tubulin as a control. (B) HepG2 cells were co-infected with the lentiviruses noted above as well as a retrovirus expressed FLAG-CPEB; western blots were probed for the FLAG epitope and tubulin. (C) HepG2 cells infected with lentivirus expressing control or CPEB shRNA were treated with insulin and western blotted for phospho (S473 and T308) and total Akt. The pAkt (Ser473) and pAkt (Thr308) data were analyzed with ANOVA (p<0.05, *). All experiments were performed 3 times. (D) HepG2 cells co-infected with the viruses noted above were western blotted for phospho (S473) and total Akt. The pAkt (Ser473) data was analyzed by ANOVA (p<0.05). (E) HepG2 cells infected with virus expressing FLAG-CPEB or FLAG-CPEBΔZF, which lacks two zinc fingers and cannot bind RNA, followed by FLAG co- immunoprecipitation and analysis of Stat3, PTEN, PDK1, IRS1, IRS2, and Socs3 RNAs by quasi-quantitative RT-PCR. Input represents 10% of total. At least 3 animals per group were used for the experiment.

The data in Figure 2 suggest that the absence of CPEB may may affect the translation of mRNAs that encode insulin-signaling proteins. To assess this possibility, we employed HepG2 human hepatocarcinoma cells that were infected with lentivirus expressing a control shRNA or one against CPEB (shCPEB). The knockdown was confirmed by RT-PCR of CPEB RNA and by down-regulation of ectopically expressed FLAG-CPEB (Figure 4A, 4B). As noted above, Akt phosphorylation was strongly reduced in *Cpeb1* KO liver following insulin injection. Depletion of CPEB from HepG2 cells also resulted in reduced insulin-stimulated Akt S473 and T308 phosphorylation (Figure 4C), although not to the extent observed in *Cpeb1* KO liver. Ectopic expression of CPEB following shRNA-mediated depletion mostly restored Akt S473 phosphorylation (Figure 4D; phospho-T308 was not examined), which further demonstrates the importance of CPEB for Akt activation. Thus, insulin-treated HepG2 cells depleted of CPEB mimic insulin-injected *Cpeb1* KO mouse liver. Finally, we expressed FLAG-tagged CPEB and CPEBΔZF, which lack two zinc fingers that are necessary for RNA binding, in HepG2 cells followed by FLAG immunoprecipitation (Figure S1C) and RT-PCR to detect putative target mRNAs. Figure 4E shows that of seven CPE-containing RNAs tested, those encoding Stat3, PTEN, PDK1, IRS1, and Pik3C (subunit of PI3-kinase) were co-immunoprecipitated with CPEB but not CPEBΔZF, suggesting that they might be direct targets of CPEB regulation.

### CPEB represses the translation of Stat3 and PTEN mRNAs

**Figure 5:**
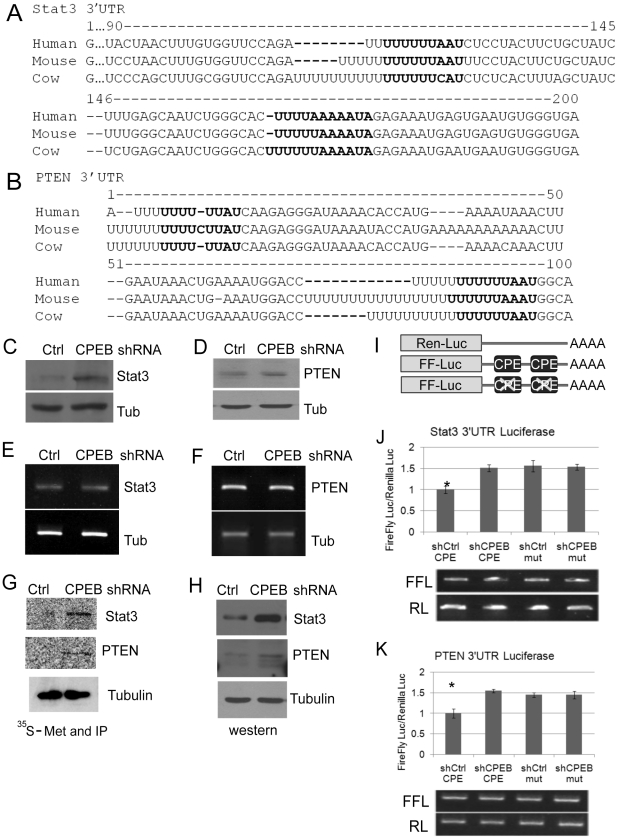
CPEB controls the synthesis of Stat3 and PTEN. (A and B) 3′ UTR sequences of Stat3 and PTEN from human, mouse and cow. The nucleotides in bold represent putative CPEs. (C and D) Western blots of Stat3 and PTEN following CPEB depletion in HepG2 cells. In panels C-H, tubulin served as a negative or input control. (E and F) Quasi-quantitative RT-PCR for Stat3 and PTEN RNAs following CPEB depletion. (G and H) HepG2 cells depleted of CPEB were pulse labeled with ^35^S-methionine for 15 min followed by Stat3, PTEN and tubulin (as a control) immunoprecipitation and SDS-PAGE analysis. These same proteins were also analyzed by western blots. (I) Representation of Renilla and firefly luciferase RNAs that were electroporated into HepG2 cells. Renilla luciferase RNA, which contained an irrelevant 3′ UTR, served as a normalization control. Firefly luciferase contained the Stat3 or PTEN 3′ UTRs as noted in panels A and B; in some cases, the CPEs in these 3′ UTRs were mutated. (J and K) The firefly and Renilla RNAs noted above were electroporated into HepG2 cells, some of which were depleted of CPEB. Firefly luciferase was normalized to the Renilla luciferase transfection control; luciferase activity derived from all RNAs was then made relative to the control shRNA. The Stat3 and PTEN data were analyzed with ANOVA; p values were 0.009 and 0.005, respectively. The asterisk refers to statistical significance (p<0.05). Data are represented as mean +/− SEM. The firefly and Renilla luciferase RNAs were also analyzed for relative stability by quasi-quantitative RT-PCR; all the RNAs had similar stabilities. At least 3 animals were used for each experiment.

Because of their central role in insulin signaling and their robust change in KO liver, we focused on PTEN and Stat3 as possible direct targets of CPEB regulation. The 3′ UTRs of both RNAs contain conserved CPEs (Figure 5A, 5B), indicating that CPEB association with these transcripts as demonstrated in Figure 4E is probably direct. Depletion of CPEB from HepG2 cells resulted in elevated amounts of Stat3 and PTEN when examined by western blotting (Figure 5C, 5D; Figure 5E and 5F show that CPEB depletion had no effect on Stat3 or PTEN mRNA levels). However, because western blots reflect steady state protein levels and not necessarily protein synthesis, control and CPEB-depleted cells were pulse-labeled with ^35^S-methionine for 15 minutes, followed by Stat3 and PTEN immunoprecipitation and SDS-PAGE analysis. Figure 5G and 5H show that de novo labeling of Stat3 and PTEN mimics the western analysis of these proteins, and indicates that their mRNAs are under direct translational control by CPEB. As a control, Figure 5G shows that ^35^S-methionine labeled tubulin was unaffected by CPEB depletion. To buttress the conclusion that CPEB regulates Stat3 and PTEN at the translational level, the 3′ UTRs of these mRNAs, containing or lacking the CPEs, were appended to firefly luciferase RNA (Figure 5I). These reporters, which together with Renilla luciferase RNA that served as an internal reference standard, were electroporated into control or CPEB-depleted HepG2 cells. The depletion of CPEB resulted in ∼50% increase of firefly luciferase RNA translation when the reporter contained either the Stat3 or PTEN 3′ UTRs. Moreover, nearly identical expression levels were observed when the CPEs were mutated, irrespective of whether the cells contained CPEB (Figure 5J, 5K, upper graph). There was no detectable difference in the stabilities of any of the electroporated RNAs (Figure 5J, 5K, lower panel). Finally, the polysome sedimentation profiles in Figure S1C show that CPEB represses translation of Stat3 and PTEN mRNAs, but not IRS1 or PDK1 mRNAs. Taken together, the data in Figure 5 as well as in Figure S1C demonstrate that CPEB acts directly on Stat3 and PTEN mRNAs to repress their translation.

### *Cpeb1* knockout mice are insulin-resistant

**Figure 6:**
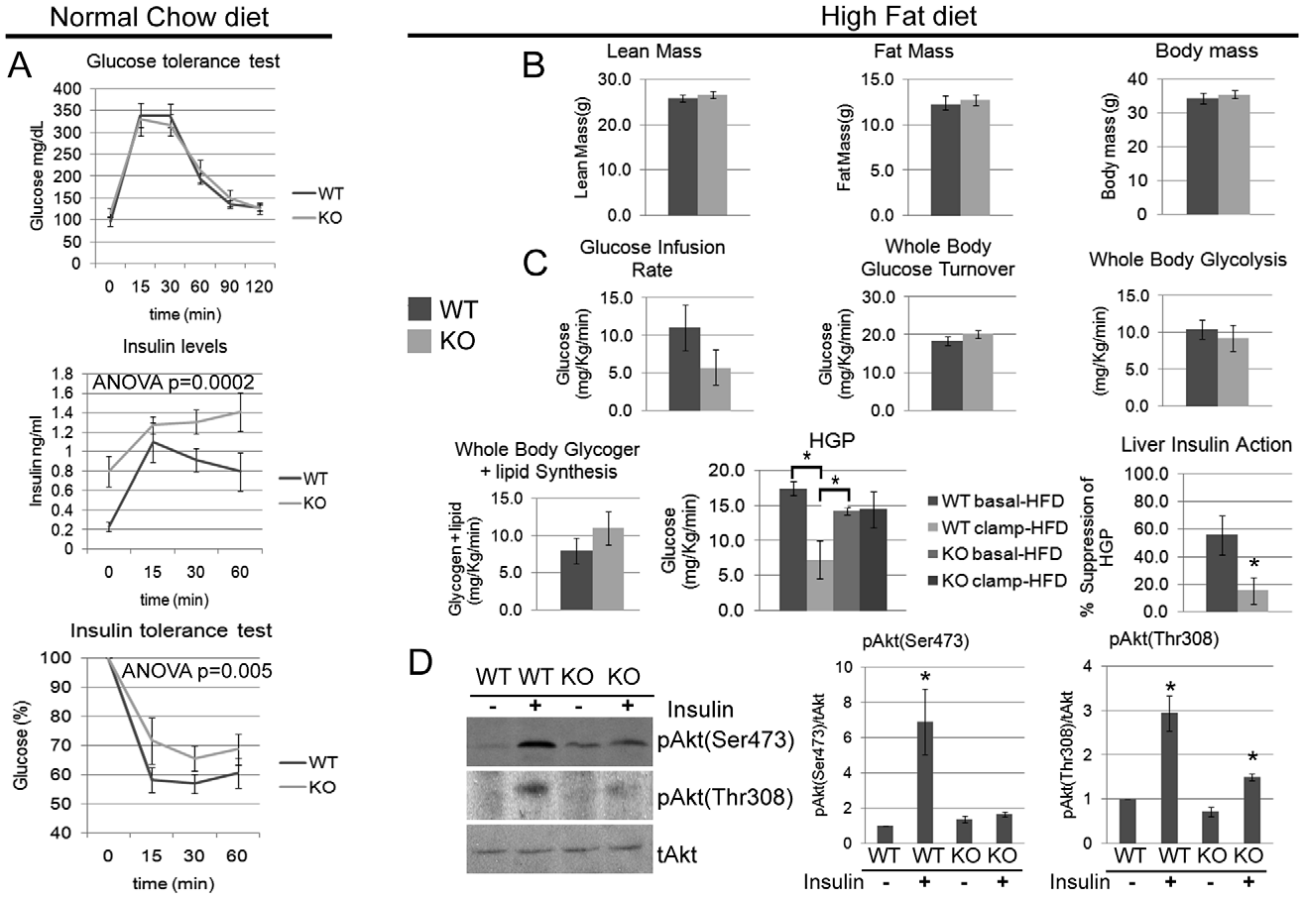
*Cpeb1* KO mice are insulin-resistant. (A) WT and KO mice were fed a normal chow diet and then examined for glucose tolerance test, serum insulin levels, and insulin tolerance. ANOVA values are as indicated in the figure. (B) Measurements for lean mass, fat mass, and total body mass of WT and *Cpeb1* KO mice fed a high fat diet. (C) Animals fed a high fat diet were subjected to euglycemic clamp analysis that determined glucose infusion rate (GIR), glucose turnover, whole body glycolysis, glycogen synthesis, hepatic glucose production (HGP), and liver insulin action (the ratio of basal to clamped HGP). The HGP data were analyzed with ANOVA with a value of 0.006. The asterisks in this panel as well as panel D refer to statistical significance (p<0.05). (D) Following the euglycemic clamp, liver proteins from WT and KO animals were probed on western blots for total and phospho-Akt (S473 and T308). The pAkt (Ser473) and pAkt (Thr308) data were analyzed with ANOVA with suggestive values of 0.01999 and 0.08335 values respectively. Data are represented as mean +/− SEM. At least 3 animals per group were used for the Western blots and at least 6 animals per group were used to measure the physiological parameters.

Although the data in Figure 2 demonstrate that the *Cpeb1* KO mice have substantial alterations in the levels of insulin signaling proteins, the animals are normal in size and mass. However, because neither of these observations speaks directly to possible changes in glucose metabolism, we subjected the animals to a glucose tolerance test (GTT), which measures glucose clearance from the blood. In this regard, the WT and KO animals were indistinguishable (Figure 6A). On the other hand, serum insulin levels measured during the GTT were significantly higher in the KO mice, which would indicate insulin resistance. To examine this possibility, we performed an insulin tolerance test (ITT). After a 5 hour fast, animals were injected with insulin, which was followed by serum glucose determination 0–60 minutes later. Compared to WT, the KO animals had significantly higher levels of glucose (Figure 6A); these data, together with those showing that insulin-injected KO animals have very low levels of phospho-Akt, indicate that the absence of CPEB induces insulin resistance. We also examined insulin secretion from isolated pancreatic islets from WT and CPEB KO mice; when challenged with glucose, we detected no statistical difference between the two groups of animals (data not shown).

We measured the levels of a number of cytokines and hormones and found that IL6 (NM_031168.1) was elevated by ∼5 fold in KO mouse serum (Figure S2A). Increased amounts of this cytokine often correlate with insulin resistance and diabetes [14]. This observation supports the notion that the KO animals have an activated inflammatory Jak-Stat signaling pathway, which is further indicated by enhanced Stat3 phosphorylation and Socs3 expression in the liver (Figure 2). Because inflammation can exacerbate insulin resistance, CPEB might control the expression of a number of molecules in several tissues to ensure that proper glucose levels are maintained.

To determine whether the insulin resistance can be magnified by metabolic insult, WT and *Cpeb1* KO mice were fed a high fat diet (HFD) for 7 weeks, which elicited obesity in both groups of animals (whole body mass); there was also no difference in fat or lean mass between the genotypes (Figure 6B). To investigate organ-specific effects of CPEB deletion on insulin action, we performed a 2-hr hyperinsulinemic-euglycemic clamp in conscious WT and KO mice. The steady-state glucose infusion rate to maintain euglycemia during clamps was reduced by ∼50% in the KO animals, although this was not statistically significant (p = 0.197). In addition, insulin-stimulated whole body glucose turnover, glycolysis, and glycogen plus lipid synthesis were unaffected in the *Cpeb1* KO mice (Figure 6C). However, hepatic insulin action, expressed as insulin-mediated percent suppression of hepatic glucose production (HGP), was significantly compromised in the *Cpeb1* KO mice (Figure 6C). The cause of this decrease in basal HGP is not clear, but because fasting levels of glucose and insulin are not affected by genotype, it may indicate, for example, aberrant gluconeogenesis. Finally, analysis of WT and KO liver following the euglycemic clamp demonstrates that although Akt was strongly phosphorylated in WT animals following insulin administration as expected, this was not the case with the *Cpeb1* KO liver (Figure 6D). We also measured fasting glucose and insulin levels in WT and KO mice fed normal chow and a high fat diet (Figure S2B). As expected, glucose levels for both genotypes increased on a high fat diet. Relative to WT, insulin levels in the CPEB KO animals were elevated on normal chow, but did not change further on a high fat diet. These data demonstrate that *Cpeb1* KO mice fed a HFD developed defects in insulin signaling and hepatic insulin resistance.

## Discussion

**Figure 7:**
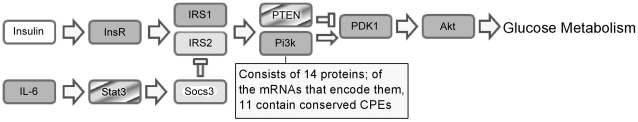
Proposed model for CPEB-dependent regulation of the insulin-signaling ribonome regulation. The dark gray boxes refer to mRNAs that contain conserved CPEs and can potentially be regulated by CPEB. The light gray boxes refer to mRNAs that contain conserved CPEs but because they are not co-immunoprecipitated with CPEB, are probably not regulated by this protein in the current settings. The striped boxes refer to mRNAs that contain conserved CPEs and are regulated by CPEB. Insulin mRNA does not contain a CPE.

Although the inflammatory response is known to be regulated by 3′UTR binding proteins that affect translation and stability [15], there is a paucity of information regarding such proteins in insulin resistance or diabetes. Our data indicate that CPEB integrates the expression of several mRNAs involved in insulin signaling. Coordinate posttranscriptional regulation of factors in a given signal transduction pathway, or coordinate regulation of multiple processing steps (e.g., splicing, export, localization, and translation) of a given RNA are two examples of a ribonome regulation (also sometimes referred to as a regulon) [16], [17]; see also [18]). Post-transcriptional regulons mediate the inflammatory response [15], and neurologic disease [19], [20]; they may do so by, for example, controlling RNA decay [15], among other events. In addition to networks mediated by RNA binding proteins, others are controlled by miRNAs. For example, miR143/145 (MI0000257/MI0000169) control the expression of several mRNAs to ensure proper smooth muscle development [21], [22]. Similarly, miR126 (MI0000153) acts on a number of RNAs to mediate angiogenesis [23]. Finally, RNA binding proteins and miRNAs can act together or oppose one another to control the expression of multiple RNAs that define complex cellular states such as neuronal differentiation or epithelial to mesenchyme transition [24]. In the examples noted above, however, no single protein or miRNA controls mRNA expression in a particular signal transduction pathway, rather, a given physiology is modulated at multiple levels (e.g., by controlling multiple signaling events) by certain proteins or miRNAs. In contrast, results reported here show that CPEB regulates the expression of several components of a single signal transduction pathway. Consequently, we propose that an insulin-signaling regulon is controlled at least in part by CPEB. It is remarkable that of the 25 components noted in Figure 7 that influence insulin signaling, mRNAs encoding 21 of them contain CPEs conserved across species. While not all of these mRNAs are likely to be directly regulated by CPEB (indeed, two of them, IRS2 and Socs3, do not co-IP with CPEB), the preponderance of CPEs suggests that CPEB influences the expression of many of them.

Although CPEB was first described as an mRNA stimulatory factor by way of inducing poly(A) tail length [25], [26], recent evidence shows that it can also repress translation [12]. Presumably, the factors with which CPEB associates in any given cell determines whether it will stimulate or repress translation. In the liver, at least for the messages we examined, CPEB seems to repress translation; when it is not present in KO mice, the synthesis of certain proteins such as PTEN and Stat3 are elevated. However, it should be borne in mind that translational repression is often reversible. It is possible that under some conditions, CPEB would be released from the RNA, which would elicit enhanced translation. Irrespective of how CPEB regulates translation, why would the insulin signaling cascade be controlled by this protein? We propose that CPEB acts as a rheostat to modulate the levels of insulin signaling proteins in response to particular environmental cues. For example, CPEB activity might be turned up or down in response to a high fat diet, and thereby modulate the degree of insulin sensitivity. If such is the case, then CPEB performance could play a central role in glucose homeostasis.

## Materials and Methods

### Animals

Male C57BL/6 *Cpeb1* KO mice (12 weeks old) (Tay and Richter, 2001) were fed normal chow diet or a high fat diet (HFD; 55% fat by calories, Harlan Teklad) for 7 weeks. Mostly littermates were used for all experiments, in some cases mice with +/−1 week of birth date were used.

### Ethics statement

The animals, which were housed in the UMass Medical School animal facility, were used according to guidelines approved the Institutional Animal Care and Use Committee and fully comply with all applicable Federal and State requirements.

### Reagents and antibodies

Mouse CPEB (WT and ΔZF) was cloned into a FLAG containing vector (Nagaoka and Richter, submitted). PTEN and Stat3 3′ UTRs (nucleotides 1–90 and 1–195, respectively) were cloned into EcoRI-XhoI sites of pcDNA3.1+ vector containing firefly luciferase. In some cases, the CPEs were mutated to C or G in place of T. *Renilla* luciferase (pRL-TK; Promega) was used as a control vector in the luciferase experiments. Antibodies to PTEN (Cell Signaling), Akt1, and pAkt473 (Cell Signaling) were a generous gift from M. Sherman. Antibodies to Socs3 (Cell Signaling) and pAkt308 (Cell Signaling) were a generous gift from R. Davis. IRS2 and IR antibody was a generous gift from M. White. Antibodies to PDK1 (Genetex), IRS1 (Upstate), tubulin (Sigma). were purchased from the indicated commercial sources. Antibodies to Stat3, pStat3, GSK-3, pGSK-3, FOXO, pFOXO, pIRS-1 were obtained from Cell Signaling. See Protocol S1 for additional information.

### Biochemical assays and cell culture

Mouse tissues (liver, fat- white adipose tissue only, muscle) and HepG2 cells were lysed in buffer (50 mM Tris-HCl (pH 7.4), 0.25 M NaCl, 1 mM MgCl_2_, 0.1 mM CaCl_2_, 1% NP-40, 0.5% deoxycholate, 0.1% SDS) with protease inhibitor (Complete, Roche) and phosphatase inhibitors (vanadate and fluoride, NEB). Western blots were performed with these samples using ECL (Perkin-Elmer), or Femto-ECL (Pierce) detection systems. RNP co-immunoprecipitation using Dynabeads (Invitrogen) was performed in the same buffer with RNase inhibitor (RNAseOut, Invitrogen) and antibody against the FLAG epitope (Sigma). The RNA was extracted from the final precipitate (or from total RNA) with Trizol (Invitrogen) and subjected to quantitative RT-PCR (Quantitect RT kit, Qiagen).

Firefly luciferase RNAs appended with Stat3 or PTEN 3′ UTRs containing or lacking CPEs were synthesized in vitro with T7 RNA polymerase (mMessage Machine kit; Ambion). Control *Renilla* luciferase RNA containing an irrelevant 3′ UTR was polyadenylated with E. coli polyA polymerase (NEB). Each of the firefly luciferase RNAs together with the *Renilla* luciferase RNA was used to transfect (with lipofectamine 2000) or nucleofect (with Amaxa nucleofector) HepG2 human hepatocarcinoma cells (ATCC #CRL-10741) that were grown to 50% confluency. Some of these cells were also infected with lentivirus harboring control or CPEB shRNA as described by Udagawa et al. (submitted). Infectivity was monitored by GFP, which was encoded by the virus. After infection, fresh DMEM with 10% FBS was added. Luciferase activity was determined 12 hr after cell transduction with a Dual-Luciferase Reporter Assay System (Promega) and normalized to the *Renilla* control. Luminescence was detected with a SAFIRE multimode microplate reader (Tecan).

### Glucose and insulin tolerance tests and plasma serum analysis

Glucose tolerance (GTT) and insulin tolerance tests (ITT) were performed using methods described previously [27]. Serum insulin concentrations during the GTT were determined using an insulin ELISA (Crystal Chem). Blood glucose was measured using an Ascensia Breeze 2 glucometer (Bayer). Statistical analysis was performed by ANOVA. Serum adipokines, cytokines, and insulin were measured by ELISA using a Luminex 200 luminometer (Millipore).

### Hyperinsulinemic-euglycemic clamp

Hyperinsulinemic-euglycemic clamps were performed at the UMass Mouse Phenotyping Center. Mice were fed normal chow or a HFD for 7 weeks. Whole body fat and lean mass were non-invasively measured using proton magnetic resonance spectroscopy (^1^H-MRS) (Echo Medical Systems). Following an overnight fast, a 2 hr hyperinsulinemic-euglycemic clamp was performed with a primed and continuous infusion of human insulin (150 mU/kg body; 2.5 mU/kg/min; Humulin; Eli Lilly), and 20% glucose was infused to maintain euglycemia (Kim et al., 2004). Whole body glucose turnover and glucose uptake in individual organs were determined by infusion of ^3^H-glucose and a bolus injection of ^14^C-2-deoxyglucose during clamps. Following the clamp, tissues were harvested for biochemical analysis [28].

### Microarray analysis

Fibroblasts (MEFs) isolated from WT and CPEB KO mice E12.5–E14.5 embryos (MEFs) were cultured in Dulbecco's Modified Eagle's Medium with 10% fetal bovine serum according to a 3T3 protocol [12]. The cells were harvested in the presence of 100 µg/ml cycloheximide (Sigma) and subjected to polysome fractionation [30]. The lysate was layered onto a continuous sucrose gradient (15%–50%) and centrifuged for 2 h at 40 000 RPM in a SW41 rotor. The RNA was isolated from polysomal fractions, pooled, and subjected to microarray analysis by UMass Medical School Genomics Core facility with Affymetrix GeneChip Mouse Genome 430A 2.0 array. The gradients were performed with biologic triplicates. The data were analyzed with DAVID Bioinformatics Resources 6.7, Panther pathway annotation [29], [32], and deposited at http://www.ncbi.nlm.nih.gov/geo/ (series number GSE28106).

### Statistical analyses

Results are presented as mean ± SEM. The error bars in all figures are SEM. A p<0.05 was considered significant. We used two-way ANOVA to assess significance (p<0.05). We further performed a pair-wise comparison (t-test) between groups.

## Supporting Information

Figure S1 CPEB mediates liver insulin signaling. (A) Western blot of CPEB in WT and KO liver. (B) Western blots of insulin signaling molecules, and tubulin as a control, in muscle and fat derived from untreated and insulin treated WT and CPEB KO mice. (C) HepG2 cells infected with virus expressing FLAG-CPEB or FLAG-CPEBΔZF, which lacks two zinc fingers and cannot bind RNA, followed by FLAG coimmunoprecipitation and Western blot analysis with anti-FLAG antibody. Input represents 10% of total. (D) Sedimentation profiles on polysome sucrose gradients of Stat3, PTEN, IRS1, and PDK1 mRNAs from WT and CPEBKO liver. The 80S monosome is indicated. RNAs were quantified by quasi-quantitative RT-PCR. Each experiment was performed with 3 different animals of each genotype.
(TIF)

Figure S2 CPEB control of IL6 and insulin levels. (A) IL6 levels in serum from WT and CPEB KO mice on a normal and high fat diet. The asterisk refers to statistical significance (p<0.05, Student's t test). (B) Glucose and insulin levels in WT and CPEB KO mice when fed normal chow (left panels) or a high fat diet (right panels). The asterisk refers to statistical significance (p<0.05, Student's t test).
(TIF)

Protocol S1 Primers for sequences used in this study.
(DOCX)

Table S1 Identification of sequences from polysomal fractions of WT and CPEB KO MEFs based on microarray analysis. Extracts from mouse embryo fibroblasts (MEFs) were centrifuged through sucrose gradients and the RNA from fractions containing polysomes was extracted and analyzed by microarrays on an Affymetrix platform.
(DOC)
