# Supplementary figures and images for: Cytoplasmic Polyadenylation Element Binding Protein Deficiency Stimulates PTEN and Stat3 mRNA Translation and Induces Hepatic Insulin Resistance
Source: PLoS Genet. 2012 Jan 12;8(1):e1002457. doi: 10.1371/journal.pgen.1002457 (PMC3257279; doi:10.1371/journal.pgen.1002457)

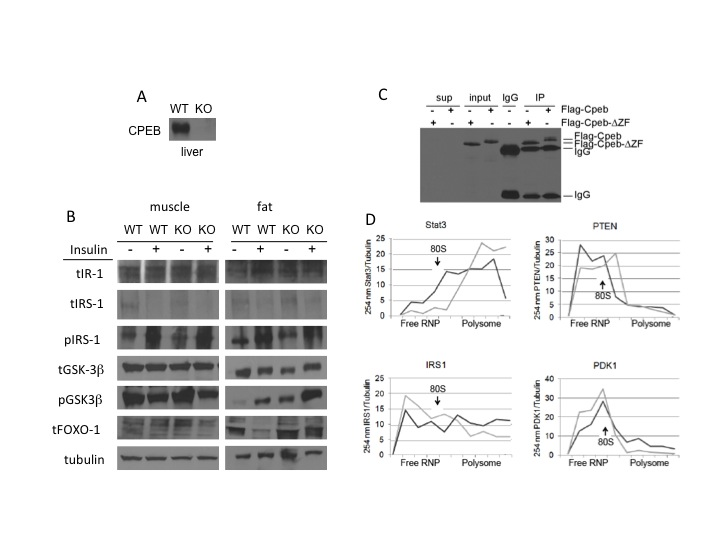

Supplement: Figure S1 — CPEB mediates liver insulin signaling. (A) Western blot of CPEB in WT and KO liver. (B) Western blots of insulin signaling molecules, and tubulin as a control, in muscle and fat derived from untreated and insulin treated WT and CPEB KO mice. (C) HepG2 cells infected with virus expressing FLAG-CPEB or FLAG-CPEBΔZF, which lacks two zinc fingers and cannot bind RNA, followed by FLAG coimmunoprecipitation and Western blot analysis with anti-FLAG antibody. Input represents 10% of total. (D) Sedimentation profiles on polysome sucrose gradients of Stat3, PTEN, IRS1, and PDK1 mRNAs from WT and CPEBKO liver. The 80S monosome is indicated. RNAs were quantified by quasi-quantitative RT-PCR. Each experiment was performed with 3 different animals of each genotype. (TIF) [file pgen.1002457.s001.tif]

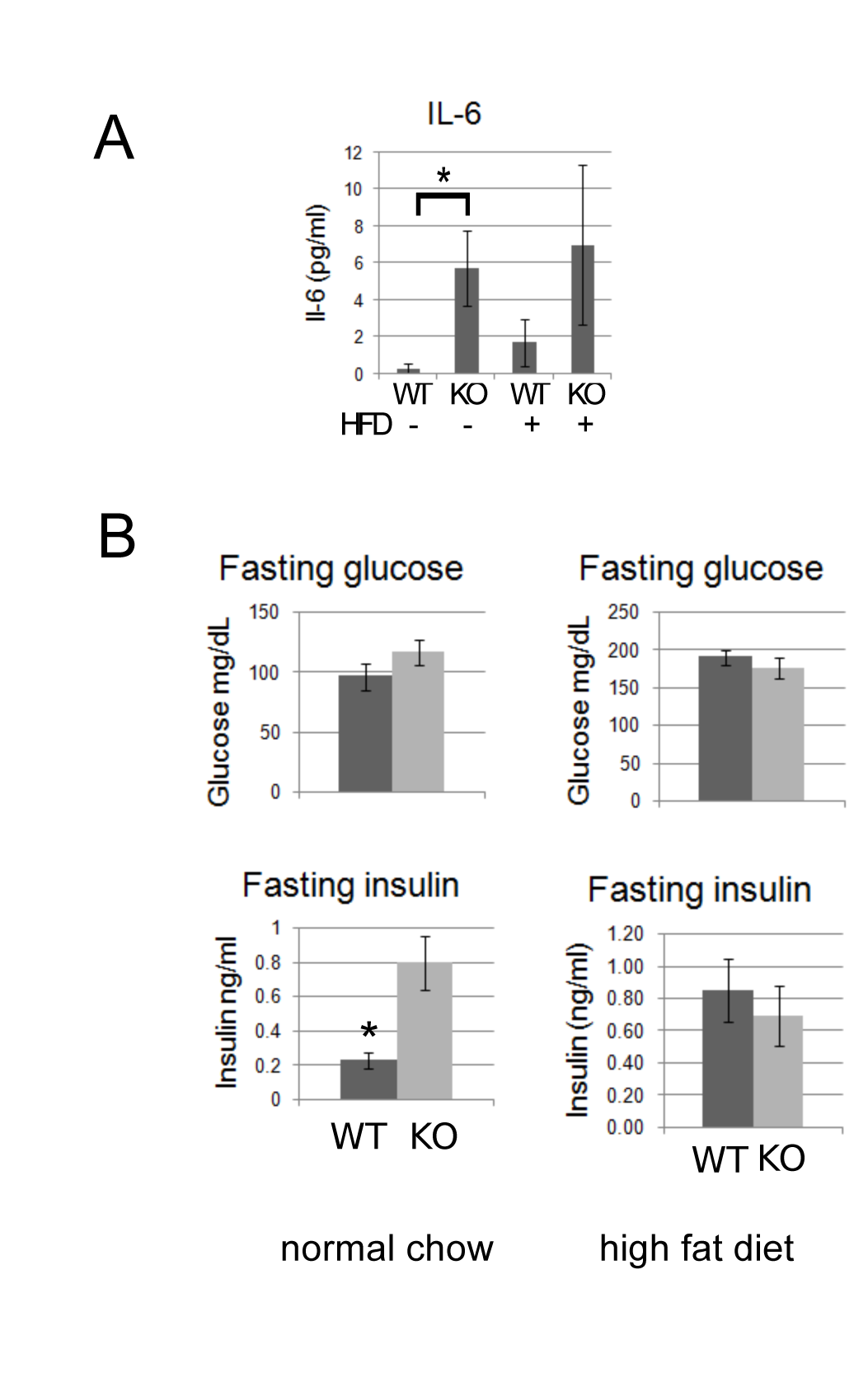

Supplement: Figure S2 — CPEB control of IL6 and insulin levels. (A) IL6 levels in serum from WT and CPEB KO mice on a normal and high fat diet. The asterisk refers to statistical significance (p<0.05, Student's t test). (B) Glucose and insulin levels in WT and CPEB KO mice when fed normal chow (left panels) or a high fat diet (right panels). The asterisk refers to statistical significance (p<0.05, Student's t test). (TIF) [file pgen.1002457.s002.tif]
