# Supplementary material for: Cytoplasmic Polyadenylation Element Binding Protein Deficiency Stimulates PTEN and Stat3 mRNA Translation and Induces Hepatic Insulin Resistance
Source: PLoS Genet. 2012 Jan 12;8(1):e1002457. doi: 10.1371/journal.pgen.1002457 (PMC3257279; doi:10.1371/journal.pgen.1002457)
Supplement: Protocol S1 — Primers for sequences used in this study. (DOCX) [file pgen.1002457.s003.docx]

**Supplemental Experimental Procedures**

Mouse primers:

PTEN-dir               ATTCCTCCAATTCAGGACCCAC         
PTEN-rev               CCTGGTATGAAGAACGTATTTACCC

Stat3-dir              TGATCGTGACTGAGGAGCTG

Stat3-rev              TGGCGGCTTAGTGAAGAAGT

Socs3-dir              GAGATTTCGCTTCGGGACTA           
Socs3-rev              GGTACTCGCTTTTGGAGCTG

Tuba1a-dir             TACCCTACCCTCGTATCCAC           
Tuba1a-rev             TGACATCTTTGGGAACCACA

IRS2-dir               GTGGAGGCTTTCCTTCAAGT           
IRS2-rev               TTTCCTGAGTGAGACATTTTCCA        

IRS1-dir               CCTCACAGTCTTCAGTGGCT           
IRS1-rev               ATAGTCCCCATTTCCTTTGC

Human primers:

Tuba1-rev              CCTCTGAAAACTCACCTTCCTCC        
Tuba1-dir              CATCAACTACCAGCCTCCCACT

 CPEB1-rev              GAAAGTCACACGACCAGAAC           
CPEB1-dir              GGTGTCCTCCCAAAGGTAAT

Stat3-dir              TGTCAGATGCCAAATGCCTG           
Stat3-rev              CTTGGTGGTGGAGGAGAACT

PTEN-dir               ATTCCTCCAATTCAGGACCCAC         
PTEN-rev               CCTGGTATGAAGAACGTATTTACCC

PTEN 3’UTR cloning into the Firefly luciferase vector:

Pcdna3.1-FFluc vector was cut by EcoR1 and Xho1 sites and an annealed oligonucleotides were phosphorylated and cloned into these sites. The oligonucleotides were as follows, on the ends (small letters) were the EcoR1 and Xho1 sites. CPE elements are surrounded by hyphens.

Forward oligo:

aattcA-TTTTTTTTTAT-CAAGAGGGATAAAACACCATGAAAATAAACTTGAATAAACTGAAAATGGACC-TTTTTTTTTTTAAT-GGCAATAGGACAc

Reverse oligo:

tcgagTGTCCTATTGCCATTAAAAAAAAAAAGGTCCATTTTCAGTTTATTCAAGTTTATTTTCATGGTGTTTTATCCCTCTTGATAAAAAAAAATg

Also the CPE elements were mutated as follows (the mutated nucleotides are in small letters):

Forward oligo:

aattcA-TTTTgTTTccT-CAAGAGGGATAAAACACCATGAAAATAAACTTGAATAAACTGAAAATGGACC-TTTTgTTTcTTcgT-GGCAATAGGACAc

Reverse oligo:

tcgagTGTCCTATTGCCACGAAGAAACAAAAGGTCCATTTTCAGTTTATTCAAGTTTATTTTCATGGTGTTTTATCCCTCTTGAGGAAACAAAATg

Stat3 3’UTR cloning into the Firefly luciferase vector:

HepG2 total cDNA was amplified for the Stat3 3’UTR with the primers containing EcoR1 and Xho1 sites:

dirStat3-3'UTR-EcoRI            ATGAATTCGGAGCTGAGAACGGAAGCTG           
revStat3-3'UTR-Xho1             ATCTCGAGTCACCCACATTCACTCATTTCTC

These were cut and cloned into the Pcdna3.1-FFluc vector.

Mutant CPE Stat3 3’UTR was cloned by amplifying the described plasmid with primers containing the forward luciferase primer:

dir-FFluc-end AGCGGTTGCCAAGAGGTTCCATCTGCCA

and

revStat3-3'UTR-Mut              ATCTCGAGTCACCCACATTCACTCATTTCTCTCTTGTTAGAAGTGCCCAGATTGCTCAAAGATAGCAGAAGTAGGAGACCAAGAAAGATCTGGA

The PCR fragment was cut by EcoR1 and Xho1 and inserted in the Pcdna3.1-FFluc vector.

The sequence of the 3’UTR is (CPE elements are surrounded by hyphens); the mutated nucleotides are in small letters)

Original 3’UTR:

GGAGCTGAGAACGGAAGCTGCAGAAAGATACGACTGAGGCGCCTACCTGCATTCTGCCACCCCTCACACAGCCAAACCCCAGATCATCTGAAACTACTAACTTTGTGGTTCCAGA-TTTTTTTTAAT-CTCCTACTTCTGCTATCTTTGAGCAATCTGGGCAC-TTTTAAAAAT-AGAGAAATGAGTGAATGTGGGTGA

Mutated 3’UTR:

GGAGCTGAGAACGGAAGCTGCAGAAAGATACGACTGAGGCGCCTACCTGCATTCTGCCACCCCTCACACAGCCAAACCCCAGATCATCTGAAACTACTAACTTTGTGGTTCCAGA-TcTTTcTTggT-CTCCTACTTCTGCTATCTTTGAGCAATCTGGGCAC-TTcTAAcAAg-AGAGAAATGAGTGAATGTGGGTGA

Cloning of Flag-CPEB-dZF:

Mouse CPEB in a pFLAG vector (Flag-CPEB) (Nagaoka K et. al. in revision) was used to clone the truncated version of CPEB lacking the Zn-finger domain. PCR fragment was amplified using the primers as follows, and cloned into AvrII and BglII sites with a stop codon in frame.

forward primer in the CPEB1 gene 5’-GCATAGAGAGAGAGGCTAGA-3’ and a reverse primer:

rev-hCPEB1dZFstopBglII          GAAGATCTCACAGAGAATCTTCTAGGTAGGGGT
