# Supplementary material for: Cytoplasmic Polyadenylation Element Binding Protein Deficiency Stimulates PTEN and Stat3 mRNA Translation and Induces Hepatic Insulin Resistance
Source: PLoS Genet. 2012 Jan 12;8(1):e1002457. doi: 10.1371/journal.pgen.1002457 (PMC3257279; doi:10.1371/journal.pgen.1002457)
Supplement: Table S1 — Identification of sequences from polysomal fractions of WT and CPEB KO MEFs based on microarray analysis. Extracts from mouse embryo fibroblasts (MEFs) were centrifuged through sucrose gradients and the RNA from fractions containing polysomes was extracted and analyzed by microarrays on an Affymetrix platform. (DOC) [file pgen.1002457.s004.doc]

**SUPPORTING INFORMATION**

**Table S1. Microarray from polysomal fractions of WT and CPEB KO MEFs**

| **Affymetrix ID** | **Gene Title** | **Gene Symbol** | **Fold Change** |
| --- | --- | --- | --- |
| 1416946_a_at | acetyl-Coenzyme A acyltransferase 1A /// acetyl-Coenzyme A acyltransferase 1B | Acaa1a /// Acaa1b | 0.77 |
| 1427943_at | acylphosphatase 2, muscle type | Acyp2 | 1.56 |
| 1425170_a_at | a disintegrin and metallopeptidase domain 15 (metargidin) | Adam15 | 0.68 |
| 1416080_at | a disintegrin and metallopeptidase domain 15 (metargidin) | Adam15 | 0.76 |
| 1448490_at | aarF domain containing kinase 4 | Adck4 | 0.77 |
| 1420619_a_at | amino-terminal enhancer of split | Aes | 0.66 |
| 1425711_a_at | thymoma viral proto-oncogene 1 | Akt1 | 0.67 |
| 1421324_a_at | thymoma viral proto-oncogene 2 | Akt2 | 0.75 |
| 1433604_x_at | aldolase 1, A isoform | Aldoa | 0.73 |
| 1451461_a_at | aldolase 3, C isoform | Aldoc | 0.74 |
| 1429246_a_at | annexin A6 | Anxa6 | 0.76 |
| 1422718_at | adaptor-related protein complex 3, sigma 2 subunit | Ap3s2 | 0.63 |
| 1418890_a_at | apolipoprotein C-IV | Apoc4 | 0.63 |
| 1420621_a_at | amyloid beta (A4) precursor protein | App | 0.71 |
| 1431429_a_at | ADP-ribosylation factor-like 4A | Arl4a | 1.39 |
| 1422609_at | cAMP-regulated phosphoprotein 19 | Arpp19 | 1.46 |
| 1419152_at | B-cell leukemia/lymphoma 10 | Bcl10 | 1.42 |
| 1426238_at | bone morphogenetic protein 1 | Bmp1 | 0.74 |
| 1424921_at | bromodomain containing 4 /// bone marrow stromal cell antigen 2 | Brd4 /// Bst2 | 0.76 |
| 1456616_a_at | basigin | Bsg | 0.67 |
| 1448505_at | nuclear DNA binding protein | C1d | 1.39 |
| 1423365_at | calcium channel, voltage-dependent, T type, alpha 1G subunit | Cacna1g | 0.77 |
| 1417229_at | calpain 1 | Capn1 | 0.7 |
| 1419583_at | chromobox homolog 4 (Drosophila Pc class) | Cbx4 | 0.77 |
| 1419473_a_at | cholecystokinin | Cck | 1.3 |
| 1417266_at | chemokine (C-C motif) ligand 6 | Ccl6 | 0.72 |
| 1448182_a_at | CD24a antigen | Cd24a | 1.43 |
| 1437502_x_at | CD24a antigen | Cd24a | 1.32 |
| 1424376_at | CDC42 effector protein (Rho GTPase binding) 1 | Cdc42ep1 | 0.77 |
| 1425455_a_at | churchill domain containing 1 | Churc1 | 1.42 |
| 1427391_a_at | procollagen, type XII, alpha 1 | Col12a1 | 0.7 |
| 1427884_at | procollagen, type III, alpha 1 | Col3a1 | 0.73 |
| 1418440_at | procollagen, type VIII, alpha 1 | Col8a1 | 0.69 |
| 1435863_at | COMM domain containing 6 | Commd6 | 1.97 |
| 1419295_at | cAMP responsive element binding protein 3-like 1 | Creb3l1 | 0.71 |
| 1416382_at | cathepsin C | Ctsc | 0.74 |
| 1422484_at | cytochrome c, somatic | Cycs | 1.31 |
| 1419994_s_at | DNA segment, Chr 10, ERATO Doi 641, expressed | D10Ertd641e | 1.46 |
| 1420368_at | density-regulated protein | Denr | 1.51 |
| 1432004_a_at | dynamin 2 | Dnm2 | 0.75 |
| 1421784_a_at | ephrin A4 | Efna4 | 0.75 |
| 1447999_x_at | glyceraldehyde-3-phosphate dehydrogenase /// similar to Glyceraldehyde-3-phosphate dehydrogenase (GAPDH) (38 kDa BFA-dependent ADP-ribosylation substrate) (BARS-38) /// predicted gene, EG432919 /// predicted gene, EG545984 /// predicted gene, EG622339 /// | EG432919 /// EG545984 /// EG622339 /// EG665036 /// EG667048 /// EG667806 /// Gapdh /// LOC384808 /// LOC632720 /// LOC639817 /// LOC670367 /// LOC671520 /// LOC671913 /// LOC672238 /// LOC676700 | 0.7 |
| 1431834_a_at | elastin microfibril interfacer 1 | Emilin1 | 0.6 |
| 1416414_at | elastin microfibril interfacer 1 | Emilin1 | 0.67 |
| 1424685_at | exosome component 4 | Exosc4 | 0.77 |
| 1418462_at | exosome component 9 | Exosc9 | 1.32 |
| 1449219_at | fatty acid desaturase 3 | Fads3 | 0.71 |
| 1417220_at | fumarylacetoacetate hydrolase | Fah | 0.6 |
| 1423407_a_at | fibulin 2 | Fbln2 | 0.77 |
| 1419486_at | forkhead box C1 | Foxc1 | 0.74 |
| 1418784_at | FXYD domain-containing ion transport regulator 7 | Fxyd7 | 0.74 |
| 1418194_at | UDP-N-acetyl-alpha-D-galactosamine:polypeptide N-acetylgalactosaminyltransferase 10 | Galnt10 | 0.76 |
| 1449974_at | glyceraldehyde-3-phosphate dehydrogenase, spermatogenic | Gapdhs | 0.68 |
| 1424150_at | glycerophosphodiester phosphodiesterase domain containing 5 | Gdpd5 | 0.76 |
| 1424300_at | gem (nuclear organelle) associated protein 6 | Gemin6 | 1.36 |
| 1424927_at | GLI pathogenesis-related 1 (glioma) | Glipr1 | 1.49 |
| 1448571_a_at | glia maturation factor, beta | Gmfb | 1.31 |
| 1439030_at | GDP-mannose pyrophosphorylase B | Gmppb | 0.67 |
| 1421947_at | guanine nucleotide binding protein (G protein), gamma 12 | Gng12 | 1.34 |
| 1429681_a_at | glycoprotein, synaptic 2 | Gpsn2 | 0.7 |
| 1418366_at | histone cluster 2, H2aa1 /// histone cluster 1, H2ad /// histone cluster 1, H2an /// histone cluster 2, H2ac /// histone cluster 2, H2aa2 | Hist1h2ad /// Hist1h2an /// Hist2h2aa1 /// Hist2h2aa2 /// Hist2h2ac | 0.74 |
| 1423906_at | heat shock factor binding protein 1 | Hsbp1 | 1.46 |
| 1435176_a_at | inhibitor of DNA binding 2 | Id2 | 4.57 |
| 1417424_at | immediate early response 3 interacting protein 1 | Ier3ip1 | 1.39 |
| 1424112_at | insulin-like growth factor 2 receptor | Igf2r | 0.76 |
| 1423996_a_at | interleukin 4 receptor, alpha | Il4ra | 0.61 |
| 1421034_a_at | interleukin 4 receptor, alpha | Il4ra | 0.72 |
| 1417887_at | integrator complex subunit 5 | Ints5 | 0.77 |
| 1417379_at | IQ motif containing GTPase activating protein 1 | Iqgap1 | 0.75 |
| 1417380_at | IQ motif containing GTPase activating protein 1 | Iqgap1 | 0.75 |
| 1417244_a_at | interferon regulatory factor 7 | Irf7 | 0.73 |
| 1418511_at | isochorismatase domain containing 2b | Isoc2b | 1.46 |
| 1423268_at | integrin alpha 5 (fibronectin receptor alpha) | Itga5 | 0.76 |
| 1432543_a_at | Kruppel-like factor 13 | Klf13 | 0.74 |
| 1455470_x_at | LIM and SH3 protein 1 | Lasp1 | 0.77 |
| 1450383_at | low density lipoprotein receptor | Ldlr | 0.75 |
| 1421462_a_at | leprecan 1 | Lepre1 | 0.77 |
| 1419042_at | LETM1 domain containing 1 | Letmd1 | 0.77 |
| 1421217_a_at | lectin, galactose binding, soluble 9 | Lgals9 | 0.75 |
| 1449874_at | lymphocyte antigen 96 | Ly96 | 1.32 |
| 1422341_s_at | lysophospholipase 3 | Lypla3 | 0.62 |
| 1427934_at | LYR motif containing 2 | Lyrm2 | 1.39 |
| 1416272_at | mitogen-activated protein kinase kinase 1 interacting protein 1 | Map2k1ip1 | 1.37 |
| 1425679_a_at | mitogen activated protein kinase 8 interacting protein 1 | Mapk8ip1 | 0.75 |
| 1415972_at | myristoylated alanine rich protein kinase C substrate | Marcks | 0.71 |
| 1449965_at | mast cell protease 8 | Mcpt8 | 1.48 |
| 1422627_a_at | McKusick-Kaufman syndrome protein | Mkks | 1.62 |
| 1423488_at | monocyte to macrophage differentiation-associated | Mmd | 1.34 |
| 1416572_at | matrix metallopeptidase 14 (membrane-inserted) | Mmp14 | 0.76 |
| 1424309_a_at | molybdenum cofactor synthesis 2 | Mocs2 | 1.46 |
| 1416380_at | Moloney leukemia virus 10 | Mov10 | 0.73 |
| 1421044_at | mannose receptor, C type 2 | Mrc2 | 0.7 |
| 1427173_a_at | mitochondrial ribosomal protein S33 | Mrps33 | 1.4 |
| 1449551_at | myosin IC | Myo1c | 0.71 |
| 1419157_at | NCK interacting protein with SH3 domain | Nckipsd | 0.6 |
| 1418996_a_at | NADH dehydrogenase (ubiquinone) 1 alpha subcomplex, 12 | Ndufa12 | 1.56 |
| 1416709_a_at | neugrin, neurite outgrowth associated | Ngrn | 0.69 |
| 1419665_a_at | nuclear protein 1 | Nupr1 | 1.62 |
| 1419666_x_at | nuclear protein 1 | Nupr1 | 1.61 |
| 1422034_a_at | paralemmin | Palm | 0.77 |
| 1424216_a_at | poly (A) polymerase alpha | Papola | 1.43 |
| 1418534_at | pyruvate dehydrogenase E1 alpha 1 | Pdha1 | 0.76 |
| 1448644_at | penta-EF hand domain containing 1 | Pef1 | 0.77 |
| 1418209_a_at | profilin 2 | Pfn2 | 1.3 |
| 1448757_at | promyelocytic leukemia | Pml | 0.77 |
| 1437845_x_at | protein O-fucosyltransferase 2 | Pofut2 | 0.71 |
| 1416573_at | protein O-fucosyltransferase 2 | Pofut2 | 0.74 |
| 1439266_a_at | polymerase (RNA) III (DNA directed) polypeptide K | Polr3k | 1.32 |
| 1422753_a_at | polymerase (RNA) III (DNA directed) polypeptide K | Polr3k | 1.3 |
| 1435697_a_at | pleckstrin homology, Sec7 and coiled-coil domains, binding protein | Pscdbp | 0.74 |
| 1425133_s_at | RAB3A interacting protein (rabin3)-like 1 | Rab3il1 | 0.77 |
| 1451643_a_at | RAB4B, member RAS oncogene family | Rab4b | 0.76 |
| 1419553_a_at | RAB geranylgeranyl transferase, b subunit | Rabggtb | 1.35 |
| 1418961_at | RCE1 homolog, prenyl protein peptidase (S. cerevisiae) | Rce1 | 0.67 |
| 1424382_at | reticulocalbin 3, EF-hand calcium binding domain | Rcn3 | 0.74 |
| 1456600_a_at | ring finger protein 7 | Rnf7 | 1.32 |
| 1453752_at | ribosomal protein L17 | Rpl17 | 1.98 |
| 1437729_at | ribosomal protein L27a | Rpl27a | 0.7 |
| 1448287_at | RNA polymerase 1-3 | Rpo1-3 | 1.37 |
| 1419460_at | ribonuclease P 14 subunit (human) | Rpp14 | 1.32 |
| 1430978_at | ribosomal protein S25 | Rps25 | 1.49 |
| 1420502_at | spermidine/spermine N1-acetyl transferase 1 | Sat1 | 1.37 |
| 1421842_a_at | secretory carrier membrane protein 4 | Scamp4 | 0.76 |
| 1453724_a_at | serine (or cysteine) peptidase inhibitor, clade F, member 1 | Serpinf1 | 0.7 |
| 1456733_x_at | serine (or cysteine) peptidase inhibitor, clade H, member 1 | Serpinh1 | 0.7 |
| 1425139_at | sestrin 2 | Sesn2 | 0.73 |
| 1436664_a_at | solute carrier family 35 (UDP-galactose transporter), member A2 | Slc35a2 | 0.73 |
| 1432533_a_at | solute carrier family 35 (UDP-galactose transporter), member A2 | Slc35a2 | 0.77 |
| 1438115_a_at | solute carrier family 9 (sodium/hydrogen exchanger), isoform 3 regulator 1 | Slc9a3r1 | 0.69 |
| 1438116_x_at | solute carrier family 9 (sodium/hydrogen exchanger), isoform 3 regulator 1 | Slc9a3r1 | 0.77 |
| 1431208_a_at | solute carrier family 9 (sodium/hydrogen exchanger), isoform 3 regulator 2 | Slc9a3r2 | 0.71 |
| 1439368_a_at | solute carrier family 9 (sodium/hydrogen exchanger), isoform 3 regulator 2 | Slc9a3r2 | 0.74 |
| 1416336_s_at | small nuclear ribonucleoprotein D1 | Snrpd1 | 1.44 |
| 1416576_at | suppressor of cytokine signaling 3 | Socs3 | 0.61 |
| 1456212_x_at | suppressor of cytokine signaling 3 | Socs3 | 0.66 |
| 1455899_x_at | suppressor of cytokine signaling 3 | Socs3 | 0.73 |
| 1436790_a_at | SRY-box containing gene 11 | Sox11 | 0.62 |
| 1450891_at | signal recognition particle 19 | Srp19 | 1.39 |
| 1426875_s_at | sulfiredoxin 1 homolog (S. cerevisiae) | Srxn1 | 1.56 |
| 1452616_s_at | single-stranded DNA binding protein 1 | Ssbp1 | 1.45 |
| 1427965_at | single-stranded DNA binding protein 1 | Ssbp1 | 1.34 |
| 1422693_a_at | SUB1 homolog (S. cerevisiae) | Sub1 | 1.41 |
| 1451005_at | SMT3 suppressor of mif two 3 homolog 1 (yeast) | Sumo1 | 1.42 |
| 1450378_at | TAP binding protein | Tapbp | 0.62 |
| 1420635_a_at | T-cell, immune regulator 1, ATPase, H+ transporting, lysosomal V0 protein A3 | Tcirg1 | 0.73 |
| 1420653_at | transforming growth factor, beta 1 | Tgfb1 | 0.75 |
| 1438769_a_at | thymocyte nuclear protein 1 | Thyn1 | 1.79 |
| 1438480_a_at | thymocyte nuclear protein 1 | Thyn1 | 1.35 |
| 1416345_at | translocase of inner mitochondrial membrane 8 homolog a1 (yeast) | Timm8a1 | 1.58 |
| 1449886_a_at | translocase of inner mitochondrial membrane 9 homolog (yeast) | Timm9 | 1.34 |
| 1451000_at | transmembrane protein 126A | Tmem126a | 1.46 |
| 1424383_at | transmembrane protein 51 | Tmem51 | 0.72 |
| 1428000_at | transmembrane protein 60 | Tmem60 | 1.34 |
| 1450731_s_at | tumor necrosis factor receptor superfamily, member 21 | Tnfrsf21 | 0.69 |
| 1425682_a_at | Tp53rk binding protein | Tprkb | 1.33 |
| 1460702_at | TP53 regulated inhibitor of apoptosis 1 | Triap1 | 1.3 |
| 1426538_a_at | transformation related protein 53 | Trp53 | 0.67 |
| 1427739_a_at | transformation related protein 53 | Trp53 | 0.71 |
| 1415978_at | tubulin, beta 3 | Tubb3 | 0.77 |
| 1451272_a_at | ubiquitin-conjugating enzyme E2F (putative) | Ube2f | 1.38 |
| 1456245_x_at | vesicle-associated membrane protein 3 | Vamp3 | 1.47 |
| 1448818_at | wingless-related MMTV integration site 5A | Wnt5a | 0.63 |
| 1454805_at | Wilms' tumour 1-associating protein | Wtap | 1.4 |
| 1424237_at | zinc finger protein 639 | Zfp639 | 1.31 |
| 1427539_a_at | ZW10 interactor | Zwint | 1.54 |
| 1427997_at | RIKEN cDNA 1110007M04 gene | 1110007M04Rik | 1.34 |
| 1417886_at | RIKEN cDNA 1810009A15 gene | 1810009A15Rik | 1.38 |
| 1430292_a_at | RIKEN cDNA 1810030N24 gene | 1810030N24Rik | 1.82 |
| 1423217_a_at | RIKEN cDNA 2510049I19 gene | 2510049I19Rik | 1.34 |
| 1428966_at | RIKEN cDNA 2610204K14 gene | 2610204K14Rik | 1.5 |
| 1452312_at | RIKEN cDNA 2810002D19 gene | 2810002D19Rik | 1.38 |
| 1423767_at | RIKEN cDNA 2810410M20 gene | 2810410M20Rik | 1.79 |
| 1426964_at | RIKEN cDNA 3110003A17 gene | 3110003A17Rik | 1.32 |
| 1453768_a_at | RIKEN cDNA 5430432M24 gene | 5430432M24Rik | 0.74 |
| 1416634_at | RIKEN cDNA 5730536A07 gene | 5730536A07Rik | 1.35 |
| 1433639_at | RIKEN cDNA 5730593F17 gene | 5730593F17Rik | 0.75 |
